# Obituary Notice

**Published:** 1867-09

**Authors:** 


### Obituary Notice.

It becomes our painful duty to announce the death of Dr. JAMES JACKSON, of Boston. He died on the 27th of August, after a protracted illness of eighteen months, in the ninetieth year of his age.

Died of nephrities, on the 23d of August, in his seventy-Second year, ALFRED ARMAND MARIE DE VELPEAU. Of humble origin, being the son of a poor blacksmith, he, by the most unflagging zeal, and the intensest ambition, overleaped every barrier obstructing his path until he reached the very pinnacle of professional eminence. "An indefatigable student, a lucid lecturer, judicious operator, and voluminous writer," Velpeau though dead, will forever live in the annals of medical history.

The submarine telegraph brings the sad intelligence of the death of the English chemist and natural philosopher, MICHAEL FARADAY, on the 27th ult., aged seventy-six.

The French lithotritist, M. CIVIALE, expired suddenly, June 13th, aged 75; and M. TROUSSEAU, "after months of cruel suffering" from cancer of the stomach, June 23d.

The German journals also announce the death of OTTO WEBER, Professor of Surgery in the University of Heidelberg, "While performing an operation of tracheotomy on a diphtheritic patient, he attempted to clear the obstructed canula by suction. Failing in it, each of his two assistants attempted the same thing and all died. Weber was only thirty-nine years of age."—*Humboldt Archives.*

SIR WILLIAM LAWRENCE died of paralysis at his residence, July 5th, at the age of eighty-three. At the time of his death he was Sergeant-Surgeon to the Queen, and Surgeon to the Royal Hospitals of St. Bartholomew and Bethlem.

The deaths of the eminent French chemist, M. PELONZE and that of M. FOLLIN, lecturer on Ophthalmology, and one of the editors of the *Archives Generales de Medicine,* are announced.

---

Prof. Hyrtl, of Vienna, received a gold medal at the last Paris Exhibition for his anatomical preparations, and Prof. Trichmann, of Cracow, the bronze for the same. Dr. Politzer, of Vienna, received an "honorable mention."

